# Supplementary material for: Activation-pathway transitions in human voltage-gated proton channels revealed by a non-canonical fluorescent amino acid
Source: eLife. 2023 Jan 25;12:e85836. doi: 10.7554/eLife.85836 (PMC9925047; doi:10.7554/eLife.85836)
Supplement: Figure 5—source code 1. [file elife-85836-fig5-code1.zip › 26-12-2022-RA-eLife-85836/DefinitionSchemeI_Fig5E.pdf]

```
#pragma rtGlobals=1      // Use modern global access method.

//          Co <--a/b-->C1 <--c/d-->C2<--e/f-->C3<--g/h-->O

Function SequentialFour(pw, tt, yw, dydt)  // four transitions five states
   Wave pw  //pw[0]= alfa1
            //pw[1]= beta1
            //pw[2]= alfa2
            //pw[3]= beta2
            //pw[4]= alfa3
            //pw[5]= beta3
            //pw[6]= alfa4
            //pw[7]= beta4
Variable tt // time variable
   Wave  yw //yw[0]=  C0
               //yw[1]=  C1
               //yw[2]=  C2
               //yw[3]=  C3
               //yw[4]=  O
   Wave dydt
   Variable B
         B = 1
   dydt[0] =                       -pw[0]*yw[0]+pw[1]*yw[1]
   dydt[1] =           -(pw[2]+pw[1])*yw[1]+pw[0]*yw[0]+pw[3]*yw[2]
   dydt[2] =           -(pw[3]+pw[4])*yw[2]+pw[2]*yw[1]+pw[5]*yw[3]
   dydt[3] =           -(pw[5]+pw[6])*yw[3]+pw[4]*yw[2]+pw[7]*yw[4]
   dydt[4] =               -pw[7]*yw[4]+pw[6]*yw[3]
End
```
