# Supplementary material for: Activation-pathway transitions in human voltage-gated proton channels revealed by a non-canonical fluorescent amino acid
Source: eLife. 2023 Jan 25;12:e85836. doi: 10.7554/eLife.85836 (PMC9925047; doi:10.7554/eLife.85836)
Supplement: Figure 5—source code 2. [file elife-85836-fig5-code2.zip › 26-12-2022-RA-eLife-85836/ProcedureToSimulateFig5E.pdf]

```
#pragma rtGlobals=1     // Use modern global access method.
function  ODEInput_SequentialFive(name, namef, nameI , numvolts)
string name
string namef
string nameI

variable numvolts
string textvolt, textpo, textvars
variable t
variable Von, Voff, Vpre, conc, normSSi
variable numptson, numptsoff, numptspre
   numptspre = 3000
   numptson = 2500
   numptsoff = 5000
variable srate // sample rate in seconds
   srate = .001// seconds
variable prestart, onstart, offstart
   prestart =0
   onstart = numptspre*srate
   offstart = numptson*srate  //;print offstart
Von = -120; Voff = -100; Vpre = -100
make /O/N = (numvolts) vt = Von+p*20, Iss, Itail, Pv, Fv
variable N, g, j, Vrev
Vrev = -120; N=8; g = .0083 //  pS x e-3 for V on mV
j = 0
make/O/N= (numptspre) temp, tempf, I0
make/O/N= (numptson) temp2, temp2f, I2
make/O/N= (numptsoff) temp3, temp3f, I3
make/O/N= (numptson+numptsoff+numptspre)  temp4, temp4f, Itot

variable D = 8// represents the effect of deltapH
make/D/O/N = 8 K
K[0] = .1*D// alf1      // rate constants in s-1 @ 0 mV
K[1] = 5// beta1
K[2] = 2*D// alfa2
K[3] = 1// beta2
K[4] = .11*D// alfa3
K[5] = 1// beta3
K[6] = 0    //open   MAKES A 4-STATE MODEL
K[7] = 0 //close  MAKES A 4-STATE MODEL

variable z1, z_1,z2, z_2,z3, z_3,zon,zoff
   z1= 0.3
   z_1= -0.3
   z2= 0.4
   z_2= -0.3
   z3= 0.8
   z_3= -0.8
   zon= 0.0
   zoff= -0.0

variable f0, f1, f2, f3,fo //fluorecence of each state

f0 = 0
```

```
f1 = 1
f2 = 2
f3 = 4
fo = 0


//------------------------------------------------------------------------------
for (j =0; j <= numvolts-1; j += 1)
t=1
do
   make/D/O/N =8 PP        //rate constants order: k1,k-1,k2,k-2,ki,k-i,kii,k-i
   PP[0] = K[0] *exp(z1*Vpre*0.04)        //alfa
   PP[1] = K[1] *exp(z_1*Vpre*0.04)       //beta
   PP[2] = K[2] *exp(z2*Vpre*0.04)        //alfa
   PP[3] = K[3] *exp(z_2*Vpre*0.04)       //beta
   PP[4] = K[4] *exp(z3*Vpre*0.04)        //alfa
   PP[5] = K[5] *exp(z_3*Vpre*0.04)       //beta
   PP[6] = K[6] *exp(zon*Vpre*0.04)       //alfa
   PP[7] = K[7] *exp(zoff*Vpre*0.04)      //beta


   Make/D/O/N=(numptspre,5) YY// wave to receive results
   SetScale/P x prestart,srate, "s", YY

   YY[0][0] = 1 // initial condition
   YY[0][1] = 0
   YY[0][2] = 0
   YY[0][3] = 0
   YY[0][4] = 0


   IntegrateODE/M=3 SequentialFour, PP, YY
   //SetScale/P x prestart,srate, "s", YY

   t=2
while (t<=1)
////////////////////////////////////////////////////////////////////////////////
do
   Make/D/O/N=(numptson,5) YYo// wave to receive results
   SetScale/P x onstart, srate,"s", YYo
   YYo[0][0] = YY[numptspre][0]  // initial condition:ON steady state
   YYo[0][1] = YY[numptspre][1]
   YYo[0][2] = YY[numptspre][2]
   YYo[0][3] = YY[numptspre][3]
   YYo[0][4] = YY[numptspre][4]



   Make/D/O/N=8 PP
   PP[0] = K[0] *exp(z1*Vt(j)*0.04)       //alfa
   PP[1] = K[1] *exp(z_1*Vt(j)*0.04)      //beta
   PP[2] = K[2] *exp(z2*Vt(j)*0.04)       //alfa
   PP[3] = K[3] *exp(z_2*Vt(j)*0.04)      //beta
   PP[4] = K[4] *exp(z3*Vt(j)*0.04)       //alfa
```

```
   PP[5] = K[5] *exp(z_3*Vt(j)*0.04)      //beta
   PP[6] = K[6] *exp(zon*Vt(j)*0.04)      //alfa
   PP[7] = K[7] *exp(zoff*Vt(j)*0.04)        //beta

   IntegrateODE/M=3  SequentialFour, PP, YYo
// SetScale/P x onstart, srate,"s", YYo

     t=3
//------------------------------------------------------------------------

while(t<=2)
do
   Make/D/O/N=(numptsoff,5) YYoo // wave to receive results
   SetScale/P x offstart, srate,"s", YYoo

   YYoo[0][0] = YYo[numptson][0]  // initial condition:ON steady state
   YYoo[0][1] = YYo[numptson][1]
   YYoo[0][2] = YYo[numptson][2]
   YYoo[0][3] = YYo[numptson][3]
   YYoo[0][4] = YYo[numptson][4]



   Make/D/O/N=8 PP
   PP[0] = K[0] *exp(z1*Voff*0.04)        //alfa
   PP[1] = K[1] *exp(z_1*Voff*0.04)       //beta
   PP[2] = K[2] *exp(z2*Voff*0.04)        //alfa
   PP[3] = K[3] *exp(z_2*Voff*0.04)       //beta
   PP[4] = K[4] *exp(z3*Voff*0.04)        //alfa
   PP[5] = K[5] *exp(z_3*Voff*0.04)       //beta
   PP[6] = K[6] *exp(zon*Voff*0.04)       //alfa
   PP[7] = K[7] *exp(zoff*Voff*0.04)      //beta

   IntegrateODE/M=3  SequentialFour, PP, YYoo
// SetScale/P x offstart, srate,"s", YYoo

     t=4
while(t<=3)
//------------------------------------------------------------------------


temp = YY[p][3] //;setscale /P x prestart,srate,"s", temp //calculate open proba

tempf = f0*YY[p][0]+f1*YY[p][1]+f2*YY[p][2]+f3*YY[p][3]+fo*YY[p][4] //calculate
 setscale /P x prestart,srate,"s", temp, tempf, I0

   I0 = temp*N*g*(Vpre-Vrev)   // current time course
// Iss[j] = temp[numptspre]//(N*g*vt(j))

temp2 = YYo[p][3]     //calculate open probability

temp2f = f0*YYo[p][0]+f1*YYo[p][1]+f2*YYo[p][2]+f3*YYo[p][3]+fo*YYo[p][4]

      setscale /P x onstart,srate,"s", temp2, temp2f, I2
```

```
   I2 = temp2*N*g*(vt(j)-Vrev)// current time course
   // -----add as leak current
   duplicate/o temp2, leak
   leak =(g*2.6)*(vt(j)-Voff)
   I2=I2+leak
   //------------------
   wavestats/Q temp2
   Iss[j] = V_max
   Pv[j]=temp2[numptson]
   Fv[j]=temp2f[numptson]

temp3 = YYoo[p][3]    //calculate open probability

temp3f = f0*YYoo[p][0]+f1*YYoo[p][1]+f2*YYoo[p][2]+f3*YYoo[p][3]+fo*YYoo[p][4]

setscale /P x offstart,srate,"s", temp3, temp3f, I3

   I3 = temp3*N*(g)*(Voff-Vrev)
   Itail[j] = temp3(offstart+.2); //print offstart
        normSSi=Itail[0]
      concatenate /O/NP {temp, temp2,temp3}, temp4
      concatenate /O/NP {tempf, temp2f,temp3f}, temp4f
      concatenate /O/NP {I0, I2, I3}, Itot

duplicate/O temp4, $name+"_"+num2str(j)
duplicate/O temp4f, $namef+"_"+num2str(j)
duplicate/O Itot, $nameI+"_"+num2str(j)

//display $nameon+"_"+num2str(j)  ; appendtograph $nameoff+"_"+num2str(j)

endfor
//sprintf textvolt, "v0=%g,v1=%g,v2=%g,v3=%g", vt[0],vt[1],vt[2],vt[3]
//sprintf textpo, "po=%g", temp[numptson]//(N*g*vt(j))
//textbox/C/N =variables textvolt
//textbox/C/N= po textpo
//sprintf textvars, "L=%g,K=%g,Kv=%g", k[4]/k[5],k[2]/k[3],k[0]/k[1]
//textbox /C/N= vars textvars
  Itail=itail/normSSi
  //print offstart+onstart
end
```
