# Supplementary material for: Activation-pathway transitions in human voltage-gated proton channels revealed by a non-canonical fluorescent amino acid
Source: eLife. 2023 Jan 25;12:e85836. doi: 10.7554/eLife.85836 (PMC9925047; doi:10.7554/eLife.85836)
Supplement: Figure 5—figure supplement 2—source code 1. [file elife-85836-fig5-figsupp2-code1.zip › 26-12-2022-RA-eLife-85836/DefinitionSchemeII_Fig5Sup2.pdf]

```
#pragma rtGlobals=1     // Use modern global access method.

//       Co <--a/b-->C1 <--c/d-->   C2  <--e/f--> C3
//                    ||           ||             ||
//             k14   || k41b   k25  || k52b     k36||k63b
//                 ||              ||             ||
//                   O4 <--g/h-->   O5   <--i/j-->O6


Function NotSoSimpleAllosteric(pw, tt, yw, dydt) // four transitions five states
            // rate constants
   Wave pw  //pw[0]= ko1
            //pw[1]= k1o
            //pw[2]= k12
            //pw[3]= k21
            //pw[4]= k23
            //pw[5]= k32
            //pw[6]= k11
            //pw[7]= k11b
            //pw[8]= k22
            //pw[9]= k22b
            //pw[10]= k33
            //pw[11]= k33b
            //pw[12]= k45
            //pw[13]= k54
            //pw[14]= k56
            //pw[15]= k65

Variable tt // time variable

               // states
   Wave  yw //yw[0]=  Co
               //yw[1]=  C1
               //yw[2]=  C2
               //yw[3]=  C3
               //yw[4]=  O1
               //yw[5]=  O2
               //yw[6]=  O3
   Wave dydt
   Variable D = 1 // allosteric coupling factor

   dydt[0] =          -pw[0]*yw[0] + pw[1]*yw[1]
   dydt[1] =          -(pw[1]+pw[2]+pw[6])*yw[1] + pw[0]*yw[0] + pw[3]*yw[2] +
   dydt[2] =          -(pw[3]+pw[4]+pw[8])*yw[2] + pw[2]*yw[1] + pw[5]*yw[3] +
   dydt[3] =          -(pw[5]+pw[10])*yw[3] + pw[4]*yw[2] + pw[11]*yw[6]
   dydt[4] =          -(pw[7]+pw[12])*yw[4] + pw[6]*yw[1] + pw[33]*yw[5]
   dydt[5] =          -(pw[13]+pw[9]+pw[14])*yw[5] + pw[12]*yw[4] + pw[8]*yw[2]
   dydt[6] =          -(pw[15]+pw[11])*yw[6] + pw[14]*yw[5] + pw[10]*yw[3]
End
```
