# Supplementary material for: Activation-pathway transitions in human voltage-gated proton channels revealed by a non-canonical fluorescent amino acid
Source: eLife. 2023 Jan 25;12:e85836. doi: 10.7554/eLife.85836 (PMC9925047; doi:10.7554/eLife.85836)
Supplement: Figure 5—figure supplement 2—source code 2. [file elife-85836-fig5-figsupp2-code2.zip › 26-12-2022-RA-eLife-85836/ProcedureToSimulate_Fig5_Sup2.pdf]

```
#pragma rtGlobals=1		// Use modern global access method.
function  ODEInput_NotSoSimpleAllosteric(name, namef, nameI , numvolts)
string name
string namef
string nameI

variable numvolts
string textvolt, textpo, textvars
variable t
variable Von, Voff, Vpre, conc, normSSi
variable numptson, numptsoff, numptspre
   numptspre = 1000
   numptson = 4500
   numptsoff = 4500
variable srate // sample rate in seconds
   srate = .001// seconds
variable prestart, onstart, offstart
   prestart = 0
   onstart = numptspre*srate
   offstart = numptson*srate  //;print offstart
Von = -100; Voff = -60; Vpre = -120
make /O/N = (numvolts) vt = -120+p*20, Iss, Itail, Pv, Fv
variable N, g, j, Vrev
Vrev = -0; N=8; g = .0083 //  pS x e-3 for V on mV
j = 0
make/O/N= (numptspre) temp, tempf, I0
make/O/N= (numptson) temp2, temp2f, I2
make/O/N= (numptsoff) temp3, temp3f, I3
make/O/N= (numptson+numptsoff+numptspre)  temp4, temp4f, Itot

variable B = .33// represents the effect of deltapH 0.7 for DpH0 12 for DpH 2
variable D = 2 // allosteric coupling

make/D/O/N = 16 K
K[0] = .1*B          // k01      // rate constants in s-1 @ 0 mV
K[1] = 1/B           // k10
K[2] = .5*B          // k12      // rate constants in s-1 @ 0 mV
K[3] = .5/B          // k21
K[4] = 2*B           // k23      // rate constants in s-1 @ 0 mV
K[5] = .0200/B          // k32   // 200 for DpH2 .02 for DpH 0
K[6] = 1*B*D         // k14
K[7] = 3/B           // k41b
K[8] = 2*B*D^2          // k25
K[9] = 3/B           // k52b
K[10] = 1*B*D^3         // k36
K[11] = 3/B          // k63b
K[12] = 01*B*D       // k45
K[13] = 3/B          // k54
K[14] = 1*B*D        // k56
K[15] = .1/B         // k65

variable z1, z_1,z2, z_2,z3, z_3,zon,zoff
   z1 = .5
   z_1 = -.3
```

```
   z2 = 1.1
   z_2 = -.4
   z3 = .6
   z_3 = -.3
   zon = .5
   zoff = -.6

variable f0, f1, f2, f3, f4, f5, f6 //fluorecence of each state

f0 = 0
f1 = 0
f2 = 0
f3 = 3
f4 = 5
f5 = 6
f6 = 6



//-----------------------------------------------------------------------------------
for (j =0; j <= numvolts-1; j += 1)
t=1
do
   make/D/O/N = 16 PP         //rate constants order: k1,k-1,k2,k-2,ki,k-i,kii,k
   PP[0] = K[0] *exp(z1*Vpre*0.04)        //alfa
   PP[1] = K[1] *exp(z_1*Vpre*0.04)       //beta
   PP[2] = K[2] *exp(z2*Vpre*0.04)        //alfa
   PP[3] = K[3] *exp(z_2*Vpre*0.04)       //beta
   PP[4] = K[4] *exp(z3*Vpre*0.04)        //alfa
   PP[5] = K[5] *exp(z_3*Vpre*0.04)       //beta
   PP[6] = K[6] *exp(zon*Vpre*0.04)
   PP[7] = K[7] *exp(zoff*Vpre*0.04)
   PP[8] = K[8] *exp(zon*Vpre*0.04)
   PP[9] = K[9] *exp(zoff*Vpre*0.04)
   PP[10] = K[10] *exp(zon*Vpre*0.04)
   PP[11] = K[11] *exp(zoff*Vpre*0.04)
   PP[12] = K[12] *exp(zon*Vpre*0.04)
   PP[13] = K[13] *exp(zoff*Vpre*0.04)
   PP[14] = K[14] *exp(zon*Vpre*0.04)
   PP[15] = K[15] *exp(zoff*Vpre*0.04)

   Make/D/O/N=(numptspre,7) YY// wave to receive results
   SetScale/P x prestart,srate, "s", YY

   YY[0][0] = 1 // initial condition
   YY[0][1] = 0
   YY[0][2] = 0
   YY[0][3] = 0
   YY[0][4] = 0
   YY[0][5] = 0
   YY[0][6] = 0


   IntegrateODE/M=3 NotSoSimpleAllosteric, PP, YY
```

```
   //SetScale/P x prestart,srate, "s", YY

   t=2
while (t<=1)
////////////////////////////////////////////////////////////////////////////
do
   Make/D/O/N=(numptson,7) YYo// wave to receive results
   SetScale/P x onstart, srate,"s", YYo
   YYo[0][0] = YY[numptspre][0]  // initial condition:ON steady state
   YYo[0][1] = YY[numptspre][1]
   YYo[0][2] = YY[numptspre][2]
   YYo[0][3] = YY[numptspre][3]
   YYo[0][4] = YY[numptspre][4]
   YYo[0][5] = YY[numptspre][5]
   YYo[0][6] = YY[numptspre][6]



   Make/D/O/N = 16 PP
   PP[0] = K[0] *exp(z1*Vt(j)*0.04)        //alfa
   PP[1] = K[1] *exp(z_1*Vt(j)*0.04)       //beta
   PP[2] = K[2] *exp(z2*Vt(j)*0.04)        //alfa
   PP[3] = K[3] *exp(z_2*Vt(j)*0.04)       //beta
   PP[4] = K[4] *exp(z3*Vt(j)*0.04)        //alfa
   PP[5] = K[5] *exp(z_3*Vt(j)*0.04)       //beta
   PP[6] = K[6] *exp(zon*Vt(j)*0.04)
   PP[7] = K[7] *exp(zoff*Vt(j)*0.04)
   PP[8] = K[8] *exp(zon*Vt(j)*0.04)
   PP[9] = K[9] *exp(zoff*Vt(j)*0.04)
   PP[10] = K[10] *exp(zon*Vt(j)*0.04)
   PP[11] = K[11] *exp(zoff*Vt(j)*0.04)
   PP[12] = K[12] *exp(zon*Vt(j)*0.04)
   PP[13] = K[13] *exp(zoff*Vt(j)*0.04)
   PP[14] = K[14] *exp(zon*Vt(j)*0.04)
   PP[15] = K[15] *exp(zoff*Vt(j)*0.04)


   IntegrateODE/M=3  NotSoSimpleAllosteric, PP, YYo
// SetScale/P x onstart, srate,"s", YYo

     t=3
//----------------------------------------------------------------------------

while(t<=2)
do
   Make/D/O/N=(numptsoff,7) YYoo // wave to receive results
   SetScale/P x offstart, srate,"s", YYoo

   YYoo[0][0] = YYo[numptson][0]  // initial condition:ON steady state
   YYoo[0][1] = YYo[numptson][1]
   YYoo[0][2] = YYo[numptson][2]
   YYoo[0][3] = YYo[numptson][3]
   YYoo[0][4] = YYo[numptson][4]
   YYoo[0][5] = YYo[numptson][5]
```

```
   YYoo[0][6] = YYo[numptson][6]


   Make/D/O/N=16 PP
   PP[0] = K[0] *exp(z1*Voff*0.04)        //alfa
   PP[1] = K[1] *exp(z_1*Voff*0.04)       //beta
   PP[2] = K[2] *exp(z2*Voff*0.04)        //alfa
   PP[3] = K[3] *exp(z_2*Voff*0.04)       //beta
   PP[4] = K[4] *exp(z3*Voff*0.04)        //alfa
   PP[5] = K[5] *exp(z_3*Voff*0.04)       //beta
   PP[6] = K[6] *exp(zon*Voff*0.04)
   PP[7] = K[7] *exp(zoff*Voff*0.04)
   PP[8] = K[8] *exp(zon*Voff*0.04)
   PP[9] = K[9] *exp(zoff*Voff*0.04)
   PP[10] = K[10] *exp(zon*Voff*0.04)
   PP[11] = K[11] *exp(zoff*Voff*0.04)
   PP[12] = K[12] *exp(zon*Voff*0.04)
   PP[13] = K[13] *exp(zoff*Voff*0.04)
   PP[14] = K[14] *exp(zon*Voff*0.04)
   PP[15] = K[15] *exp(zoff*Voff*0.04)


   IntegrateODE/M=3  NotSoSimpleAllosteric, PP, YYoo
// SetScale/P x offstart, srate,"s", YYoo

     t=4
while(t<=3)
//----------------------------------------------------------------------------


temp = YY[p][4]+YY[p][5]+YY[p][6] //;setscale /P x prestart,srate,"s", temp

tempf = f0*YY[p][0]+f1*YY[p][1]+f2*YY[p][2]+f3*YY[p][3]+f4*YY[p][4]+f5*YY[p][5]+
 setscale /P x prestart,srate,"s", temp, tempf, I0

   I0 = temp*N*g*(Vpre-Vrev)   // current time course
// Iss[j] = temp[numptspre]//(N*g*vt(j))

temp2 = YYo[p][4]+YYo[p][5]+YYo[p][6]      //calculate open probability

temp2f = f0*YYo[p][0]+f1*YYo[p][1]+f2*YYo[p][2]+f3*YYo[p][3]+f4*YYo[p][4]+f5*YYo

      setscale /P x onstart,srate,"s", temp2, temp2f, I2

   I2 = temp2*N*g*(vt(j)-Vrev)// current time course
   wavestats/Q temp2
   Iss[j] = V_max
   Pv[j]=temp2[numptson]// open probability
   Fv[j]=temp2f[numptson]

temp3 = YYoo[p][4]+YYoo[p][5]+YYoo[p][6]   //calculate open probability

temp3f = f0*YYoo[p][0]+f1*YYoo[p][1]+f2*YYoo[p][2]+f3*YYoo[p][3]+f4*YYoo[p][4]+f
```

```
setscale /P x offstart,srate,"s", temp3, temp3f, I3

   I3 = temp3*N*(g/2)*(Voff-Vrev)
   Itail[j] = temp3(offstart+.2); //print offstart
        normSSi=Itail[0]
      concatenate /O/NP {temp, temp2,temp3}, temp4
      concatenate /O/NP {tempf, temp2f,temp3f}, temp4f
      concatenate /O/NP {I0, I2, I3}, Itot

duplicate/O temp4, $name+"_"+num2str(j)
duplicate/O temp4f, $namef+"_"+num2str(j)
duplicate/O Itot, $nameI+"_"+num2str(j)

//display $nameon+"_"+num2str(j)  ; appendtograph $nameoff+"_"+num2str(j)

endfor
//sprintf textvolt, "v0=%g,v1=%g,v2=%g,v3=%g", vt[0],vt[1],vt[2],vt[3]
//sprintf textpo, "po=%g", temp[numptson]//(N*g*vt(j))
//textbox/C/N =variables textvolt
//textbox/C/N= po textpo
//sprintf textvars, "L=%g,K=%g,Kv=%g", k[4]/k[5],k[2]/k[3],k[0]/k[1]
//textbox /C/N= vars textvars
  Itail=itail/normSSi
  //print offstart+onstart
end
```
