# Supplementary material for: Activation-pathway transitions in human voltage-gated proton channels revealed by a non-canonical fluorescent amino acid
Source: eLife. 2023 Jan 25;12:e85836. doi: 10.7554/eLife.85836 (PMC9925047; doi:10.7554/eLife.85836)
Supplement: Peer review history

# Editor report: Editor's evaluation

**DOI:** 10.7554/eLife.85836.sa0

**Authors:** Stephan A Pless

**Affiliations:** https://ror.org/035b05819 University of Copenhagen Denmark

This work by Suarez-Delgado et al. employs the fluorescent non-canonical amino acid Anap to explore the mechanisms by which the Hv1 proton channel is modulated by voltage and pH. Two kinetically distinct fluorescence components support the presence of at least three conformational states in the activation pathway of Hv1 and measurements using different pH gradients suggest that voltage sensor movement and channel opening are similarly affected by pH gradients. The work provides a rigorous and thorough characterization of how Anap can be used to explore mechanisms of gating and regulation and will be of interest to physiologists and biophysicists interested in mechanistic studies of membrane proteins using non-canonical fluorescent amino acids.

# Decision letter

**DOI:** 10.7554/eLife.85836.sa1

**Affiliations:** https://ror.org/035b05819 University of Copenhagen Denmark

In the interests of transparency, eLife publishes the most substantive revision requests and the accompanying author responses.

[Editors' note: this paper was accepted for publication by *eLife* after peer review and endorsement by Biophysics Colab.]
